# Mutations of SARS-CoV-2 RBD May Alter Its Molecular Structure to Improve Its Infection Efficiency

**DOI:** 10.3390/biom11091273

**Published:** 2021-08-25

**Authors:** Ahmed L. Alaofi, Mudassar Shahid

**Affiliations:** Department of Pharmaceutics, College of Pharmacy, King Saud University, P.O. Box 2457, Riyadh 11451, Saudi Arabia; mahmad1@ksu.edu.sa

**Keywords:** wild-type RBD, mutant RBDs, SARS-CoV-2, molecular dynamics simulations, RBD flexibility, principle component analysis, free energy landscape

## Abstract

The receptor-binding domain (RBD) of severe acute respiratory syndrome coronavirus 2 (SARS-CoV-2) mediates the viral–host interaction and is a target for most neutralizing antibodies. Nevertheless, SARS-CoV-2 RBD mutations pose a threat due to their role in host cell entry via the human angiotensin-converting enzyme 2 receptor that might strengthen SARS-CoV-2 infectivity, viral load, or resistance against neutralizing antibodies. To understand the molecular structural link between RBD mutations and infectivity, the top five mutant RBDs (i.e., N501Y, E484K L452R, S477N, and N439K) were selected based on their recorded case numbers. These mutants along with wild-type (WT) RBD were studied through all-atom molecular dynamics (MD) simulations of 100 ns. The principal component analysis and the free energy landscape were used too. Interestingly, N501Y, N439K, and E484K mutations were observed to increase the rigidity in some RBD regions while increasing the flexibility of the receptor-binding motif (RBM) region, suggesting a compensation of the entropy penalty. However, S477N and L452R RBDs were observed to increase the flexibility of the RBM region while maintaining similar flexibility in other RBD regions in comparison to WT RBD. Therefore, both mutations (especially S477N) might destabilize the RBD structure, as loose conformation compactness was observed. The destabilizing effect of S477N RBD was consistent with previous work on S477N mutation. Finally, the free energy landscape results showed that mutations changed WT RBD conformation while local minima were maintained for all mutant RBDs. In conclusion, RBD mutations definitely impact the WT RBD structure and conformation as well as increase the binding affinity to angiotensin-converting enzyme receptor.

## 1. Introduction

The pandemic of coronavirus disease 2019 (COVID-19) has infected over 173 million individuals (at the time of this writing) and caused millions of deaths around the globe [1]. Severe acute respiratory syndrome coronavirus 2 (SARS-CoV-2), responsible for COIVD-19, is a single-stranded positive-strand RNA virus that belongs to the Coronaviridae family [2]. Coronaviruses (CoVs) were previously known to be present in the environment and to infect humans, although the earlier infections resulted in mild symptoms and were limited to local areas. However, deadly human CoVs, such as SARS-CoV and Middle East respiratory syndrome coronavirus (MERS-CoV) as well as SARS-CoV-2, have appeared in the past two decades. These CoVs are more severe and cover more ground in every passing phase, as they can cause deadly pneumonia in humans along with other gastrointestinal diseases [3,4,5]. SARS-CoV-2 is characterized by efficient transmission and its ability to rapidly spread worldwide despite its lower mortality rate (3.3%) in comparison to SARS-CoV (10%) and MERS-CoV (37%) [6].

Structural components of SARS-CoV-2 have been extensively studied [7,8,9,10,11]. On mature virus, the spike (S) glycoprotein on the surface of SARS-CoV-2 is composed of an extracellular domain (EC), transmembrane (anchor) domain, and short intracellular tail domain (IC) [11,12]. EC domain has two functional subunits: a receptor-binding subunit (S1) and a membrane-fusion (S2) subunit [13]. The host cell (cellular) proteases cleave S protein at the boundary between S1–S2 site and S2′ site during host–virus membrane fusion [11,14]. Further, the S1 subunit compromises the receptor-binding domain (RBD) that is essential for receptor binding and contributes to stabilizing the S2 subunit that harbors the fusion machinery. After the S1 subunit binds to the cellular receptor, subsequent structural rearrangements of metastable S glycoprotein occur to allow fusion between the viral and the host cell membranes. The structural rearrangements can be explained by the conformational dynamics behavior of the S glycoprotein trimer that eventually results in an open (standing) conformation in order to successfully achieve binding and fusion events [7]. In fact, S glycoprotein is a target for immune cells that neutralize the virus, as many vaccines have been developed based on the antigenicity of S glycoprotein. SARS-CoV-2 RBD is known to bind to human angiotensin converting enzyme 2 (AEC2), specifically through the receptor-binding motif (RMB) of RBD, to mediate the viral–host interaction. Moreover, the RBM bears a flexible nature and contains most of the SARS-CoV-2 RBD residues that bind directly to ACE2 receptor [10]. However, RBD must adopt a specific conformation (up conformation) to bind efficiently to ACE2 [15].

Importantly, the genetic mutation of SARS-CoV-2 might be linked to the viral properties that influence the viral transmission mode and severity of COVID-19 as well as RBD conformation [15,16]. One of the dominant variants during COVID-19 pandemic has been the D614G mutation (not in the RBD region) of S glycoprotein; several reports have claimed that this mutation is able to increase the infectivity and stability of SARS-CoV-2 [17,18,19,20,21]. Up until now, most neutralizing antibodies against SARS-CoV-2 have been targeting its RBD [22,23,24,25,26]. However, there have been several mutations reported in SARS-CoV-2 RBD, such as N501Y, L452R, S477N, E484K, A502S, N439K, S494P, T478K, K417N, and K417T. These mutations pose a threat due to their role in host cell entry via the hACE2 receptor, which might strengthen SARS-CoV-2 infectivity, conformation and stability of RBD, viral load, or resistance against neutralizing antibodies [27,28,29,30,31]. For instance, according to a recent report, the N439K variant showed resistance against several neutralizing antibodies, including one authorized by the U.S. Food and Drug Administration (FDA) for emergency use [32]. It is clear that these mutation sites are mostly located in the RBM in the RBD region of SARS-CoV-2, which has shown a flexible nature. Importantly, the molecular dynamics and flexibility of the RBD region might have contributed to SARS-CoV-2 infectivity [31,32]. The main aim of this study was to assess the effects of critical RBD mutations on its molecular structural characterization. Therefore, in this study, mutant RBDs of SARS-CoV-2 were selected from the RBD mutation tracker website (CovMT) [33]; the following variants were selected: N501Y, L452R, S477N, N439K, and E484K. The CovMT website ranks mutant RBDs based on their recorded case numbers, hence, we selected the mutant RBDs with the highest recorded case numbers. Then we investigated the flexibility, conformational changes, principal component analysis (PCA), and free energy landscape of these mutant RBDs along with wild-type (WT) RBD via utilizing molecular dynamics (MD) simulations.

## 2. Materials and Methods

### 2.1. RBD Selection and Structure Preparation

The mutant RBDs selection was based on the RBD mutation tracker website (CovMT). The CovMT website was used to select the mutant RBDs that showed the highest recorded case numbers from February until May 2021 [34]. For RBD structure preparation, the X-ray structures (starting structures) were downloaded from Protein Data Bank for WT RBD (PDB ID: 6M0J) and N501Y mutant (PDB ID: 7NEG) [10,34]. For other mutant RBDs, we mutated the original sequence of WT RBD with a single mutation L452 to R, S477 to N, N439 to K, or E484 to K. Afterward, the sequences were uploaded to Iterative Threading Assembly Refinement (I-TASSER) platform to model the starting structures for the L452R, S477N, N439K, and E484K mutants before MD simulations [35]. The modeled structures were validated using an online RAMPAGE server for Ramachandran plot analysis [36], Verify 3D [37], and ProSA analysis [38] (Supplementary Figures).

### 2.2. Simulation Parameters

MD simulations were performed using GROMACS 5.1.4 program with CHARMM27 force field [39,40]. The starting coordinates for each MD simulation system were either X-ray structures (i.e., for the WT RBD and N501Y mutants) or the modeled structures for the L452R, S477N, N439K, and E484K mutants. Then, we performed MD simulations following our previous work with minor changes [41,42]. Each system was solvated with TIP3P water [43] with the minimal distance of 1.0 nm between the solute and the wall of the dodecahedron box. Ionization states were assigned to titratable residues corresponding to pH 7.0 condition. A proper amount of Na and Cl ions were added instead of water molecules to imitate an ionic strength of 0.15 M. Afterward, a brief energy minimization was performed using the steepest descent algorithm, followed by 20-ps-long MD simulations with positional restraints on all heavy atoms. Then, a 100-ps-long unconstrained equilibration MD simulation was done at a constant temperature (300 K) and pressure using Berendsen and Parrinello–Rahman coupling methods, respectively. Pressure coupling was performed using a reference pressure of 1.0 bar and a time constant of 1.0 ps. Finally, a 100-ns-long production MD simulation at a constant temperature of 300 K, maintained by the v-rescale thermostat, was performed [41,42,44].

### 2.3. Visualization and Analysis

The analysis of output structures from 100-ns MD simulations was performed by the following GROMACS commands: *gmx rmsf* to calculate root mean square fluctuation (RMSF) values; *gmx rmsd* to calculate root mean square deviation (RMSD) values; *gmx gyrate* to calculate the radius of gyration; *gmx sasa* to calculate the solvent accessible surface area; *gmx hbond* to monitor hydrogen bonds during the simulations; *gmx covar* and *gmx anaeig* to calculate PCA, *gmx sham* to obtain free energy landscape from PCA analysis in GROMACS utilities, and finally *xmgrace* to depict the plots. PyMol was used to visualize and represent all RBD structures and to depict Porcupine plot (Sean M. Law et al.). The Dictionary of Protein Secondary Structure (DSSP) program with *gmx do_dssp* was used to monitor the secondary structures during the 100-ns MD simulations [45].

## 3. Results

### 3.1. RMSD

**Figure 1:**
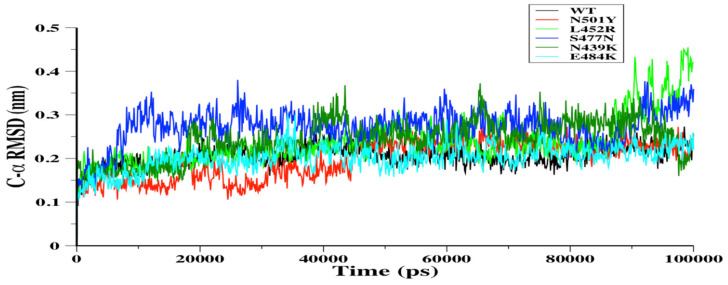
The C-α root mean square deviation (C-α RMSD) in nm was depicted for wild-type (WT) (black), N501Y (red), L452R (light green), S477N (blue), N439K (green), and E484K (cyan) RBDs during the 100-ns MD simulations.

C-α root mean square deviation (C-α RMSD) was assessed during the 100-ns MD simulation runs for WT RBD and N501Y, L452R, S477N, N439K, and E484K RBDs systems (Figure 1). There were no significant differences between WT and mutant RBDs systems during the simulations, thus indicating stable MD simulations for WT and mutant RBDs systems.

### 3.2. Mutation Effects on the RBD Flexibility

**Figure 2:**
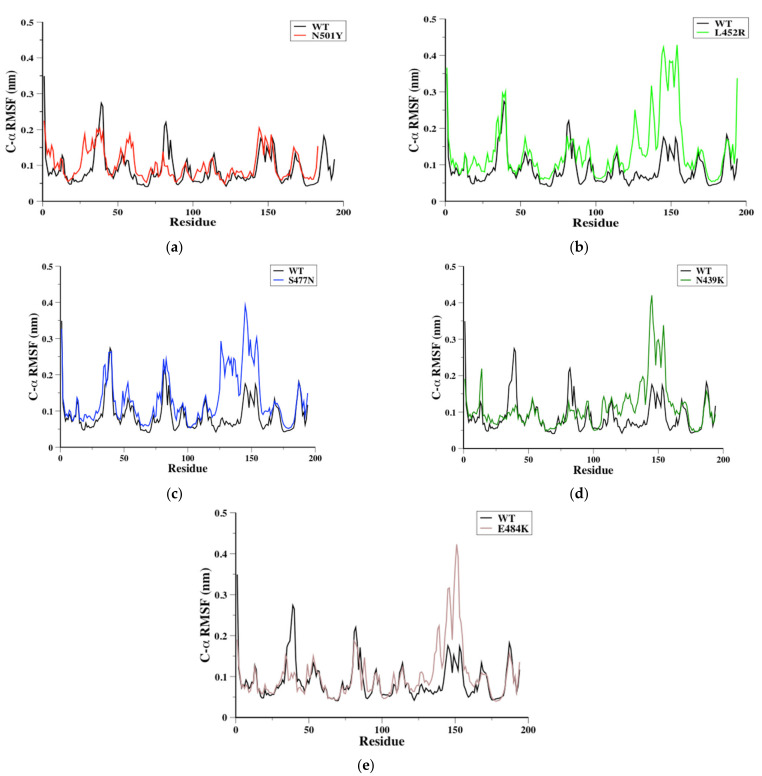
The C-α root mean square fluctuation (C-α RMSF) in nm for WT RBD aligned with either N501Y (**a**), L452R (**c**), S477N (**d**), N439K (**e**), or E484K (**f**) RBDs as a function of RBD residues obtained from the 100-ns MD simulations. The WT RBD cartoon representative (**b**) was plotted to indicate the RBD regions of interest.

The flexibility of SARS-CoV-2 RBD residues might be crucial in identifying potential binding sites during RBD-ACE2 protein–protein interaction (PPIs) [32,46,47,48]. Therefore, C-α root mean square fluctuation (C-α RMSF) was obtained in order to evaluate the flexibility during MD simulations for each system (Figure 2). To facilitate the flexibility comparison of the mutant RBDs to WT RBD, we compared the flexibility of four distinctive RBD regions named S366-S371, P384-D389, P412-D428, and Y473-C489 (the latter is located in RBM) based on the original WT RBD sequence (Figure 2a). Interestingly, our results showed that RBD mutations can increase or rigidify some parts of RBD flexibility based on the mutation site and type. The results showed that the S366-S371 has similar flexibility to L452R and S477N compared to WT RBDs, and that this domain was significantly rigid in N439K and E484K RBDs and slightly rigid in N501Y RBD compared to WT RBD (Figure 2). In the loop P384-D389, the flexibility was increased only in N501Y RBD among other mutants (Figure 2a). For L452R, S477N, and E484K RBDs, a similar flexibility was observed in the loop P412-D428 compared to WT RBD, while N501Y and N439K showed more rigid structures in the same domain (Figure 2). Finally, the loop Y473-C489 of N501Y RBD showed similar flexibility to WT RBD, while the other mutant RBDs showed a significant increase in the same domain flexibility (Figure 2). It is worth mentioning that the loop Y473-C489 is located in the RBM of SARS-CoV-2 RBD and showed high flexibility, which is in agreement with previously reported results [9,32,49].

### 3.3. RBM Loops Characterization

**Figure 3:**
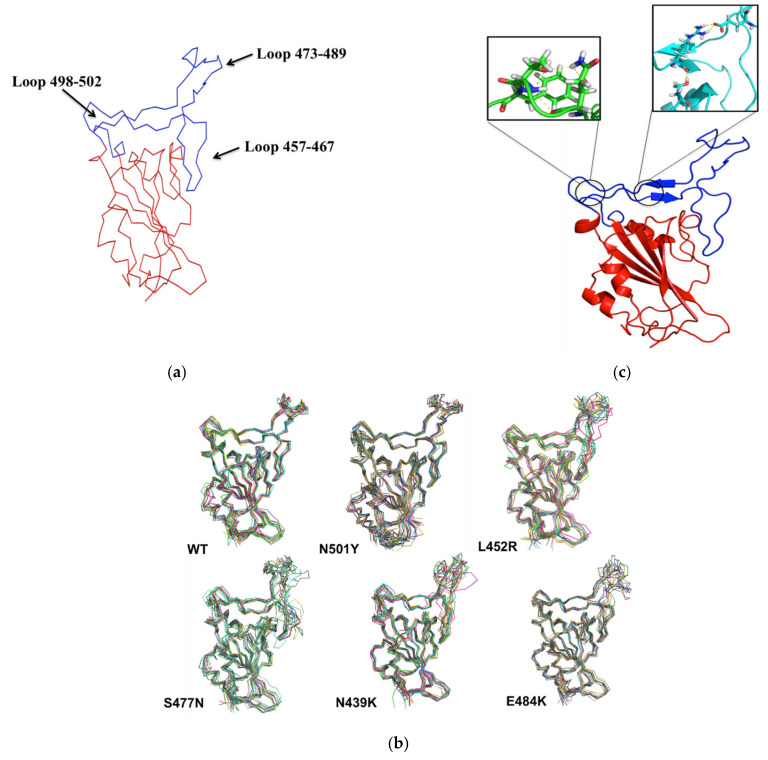
To illustrate RBD region, the ribbon representative of 100 ns conformer of WT RBD is depicted with red color and RBM is colored in blue and black arrows indicate RBM loop regions (**a**). Ten conformers obtained every 10 ns were aligned together from each MD simulations system. The ribbon representative for WT, N501Y, L452R, S477N, N439K, or E484K RBDs was aligned together in order to observe the conformational changes of RBD regions during the simulations (**b**). A cartoon representative was plotted to show the hydrogen bonds formed by the N501Y and L452R mutations; small boxes provide enlarged images of the hydrogen bonds formed for N501Y (green) and L452R (cyan) RBDs (**c**).

The RBM region (438–510) of SARS-CoV-2 RBD encompasses residues that directly bind to ACE2 receptor, as mentioned above. RBM region have mainly loop structures, and analyses of these structures might provide an indicator for RBM’s favorable binding conformation(s) (Figure 3a). Therefore, trajectories from each 100-ns MD simulation system were analyzed to evaluate the RBM loops conformation of WT, N501Y, L452R, S477N, N439K, and E484K RBDs. Then, 10 conformers from each simulation system were extracted (i.e., every 10 ns) and aligned together (Figure 3b). Clearly, N501Y was the only mutant RBD that featured RBM conformers that varied significantly at loop 498–502 (slightly flexible) during the 100-ns MD simulations (Figure 3b). This loop of RBM did not vary in the other mutant RBDs (especially in E484K RBD) (Figure 3b). Additionally, S477N and L452R showed different loop conformers at loop 457–467 (a flexible loop) of RBM, while N501Y, E484K, and N439K showed similar conformers in the same region during the simulations (Figure 3b). Moreover, RBM loop conformers at the Y473-C489 domain (a flexible domain) were changed significantly during the 100-ns MD simulations for WT RBD and all mutant RBDs (Figure 3b).

### 3.4. 3D Conformational Analysis

**Figure 4:**
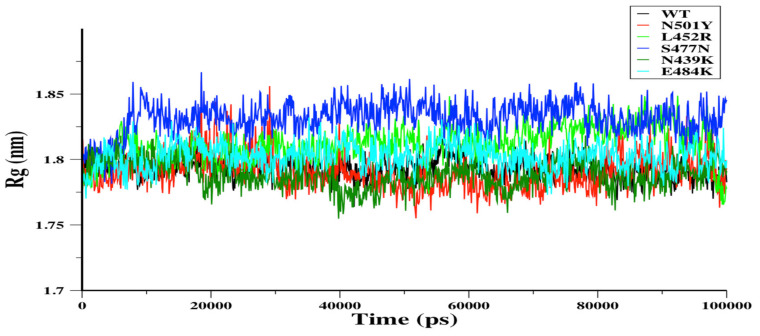
The radius of gyration (Rg) in nm was plotted versus simulation time (ps). WT (black), N501Y (red), N439K (green), and E484K (cyan) RBDs showed a relatively similar Rg for all of the simulations (**a**). L452R (light green) and S477N (blue) were different from WT RBD over most of the 100-ns simulations. However, L452R showed similar conformations in the last frame of MD simulations.

The radius of gyration (Rg) can be used to assess the conformational compactness of proteins. Rg for WT and its mutants N501Y, L452R, S477N, N439K, and E484K were assessed during the 100-ns MD simulations (Figure 4). Rg assessment was done for a residue range of 334–516 because the starting (X-ray) structure of N501Y RBD was missing six residues of C-terminus and one residue of N-terminus compared to the original WT (i.e., the total WT residues is 333–522). Other mutant RBDs were modeled using I-TASSER platform based on the WT sequence. For each RBD system, the Rg of N501Y, L452R, N439K, and E484K RBDs showed similar conformational compactness over the 100-ns MD simulations, while S477N RBD showed a significantly looser conformation in comparison to WT RBD (Figure 4a). The solvent accessible surface area (SASA) can be used to predict conformational changes due to mutations and protein–protein interactions [50]. Therefore, the SASA was calculated to measure the interaction between RBDs and solvent molecules. The SASA of WT, N501Y, and N439K RBDs showed similar profiles, and both L452R and E484K showed slight increases in their SASA. However, the SASA of S477N RBD increased significantly in comparison to the WT RBD (Figure S1). This was consistent with the radius of gyration analysis, as the loose conformation was observed in S477N RBD.

### 3.5. Changes in WT RBD Secondary Structure

The secondary structure changes were monitored during the 100-ns MD simulations as well as the changes in flanking residues for each mutation site. The total number of secondary structures did not change, indicating a stable structure for the mutants (Figure S2). The residue range 382–392 tended to form α-helix structure in N501Y and S477N RBDs in comparison to a turn structure in WT. The E484K RBD showed significant increase in α-helix structure in comparison to both the N501Y and S477N RBDs (Figure S3A,C). Moreover, an α-helix structure was observed in the residue range 417–422 of the N501Y, L452R, and S477N RBDs (Figure S3A–C). The turn structure of WT RBD was changed to a bend structure in the residue range 482–487 of the S477N, N439K, and E484K RBDs (Figure S3C,E,F). Furthermore, the α-helix structure of WT RBD became a turn structure in both the N439K and E484K RBDs (Figure S3D). Finally, we monitored the changes in the secondary structures of flanking residues near mutation sites in the RBDs. Only residues around Glu484 site (i.e., in E484K RBD) were changed from α-helix to turn structure. There were no observed changes in the secondary structures near mutation sites for other RBDs

### 3.6. Hydrogen Bonds Monitoring

Hydrogen bonds (H-bonds) are a major stabilizing force in protein tertiary structures. The RBD mutations could affect the molecular dynamics and therefore improve the viral binding affinity, which can be affected by H-bonds. Our results showed that only the mutation of N501Y and L452R formed strong hydrogen bonds (3.0 Å), and a weak hydrogen bond was observed in N439K with neighboring residues (Figure 3c). As anticipated, the mutation of S477N and E484K would not form hydrogen bonds since their mutation sites are located in the binding interacting loop (Figure 2b). This was consistent with the low H-bond number observed in both the S477N and E484K RBDs during the 100-ns MD simulations (Figure S4). Other RBDs (i.e., N501Y, L452R, and N439K) showed similar H-bond numbers to WT RBD, at least after 80 ns of MD simulations.

### 3.7. Principal Component Analysis

Principal component analysis (PCA) has been extensively used to study the influence of residue mutations on proteins essential dynamics during simulations [49,51,52,53,54,55]. The large-scale dynamics are often related to the biological function of proteins. Hence, PCA is used to reduce the number of dimensions required to describe protein dynamics. The protein dynamics are confined within a few principal component (PC) modes, usually PC1 and PC2, that are presumably meaningful to biological functions. Projection of the simulation trajectories on these eigenvectors, which mostly have the largest eigenvalues, can define the essential subspace in which protein dynamics occur [56].

**Figure 5:**
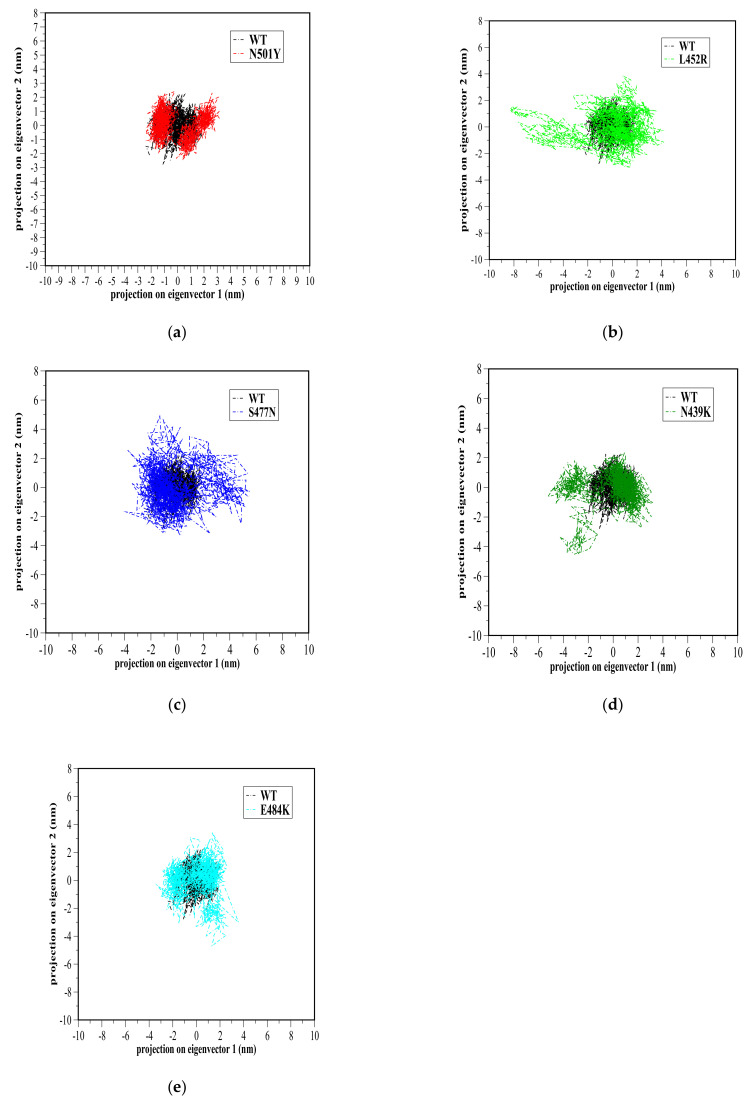
Projection of the motion of WT RBD (black color) aligned with either (**a**) N501Y (red color), (**b**) L452R (light green color), (**c**) S477N (blue color), (**d**) N439K (green color), or (**e**) E484K (cyan color) mutant receptor-binding domains (RBDs) along with the first two principal eigenvectors in nm.

**Figure 6:**
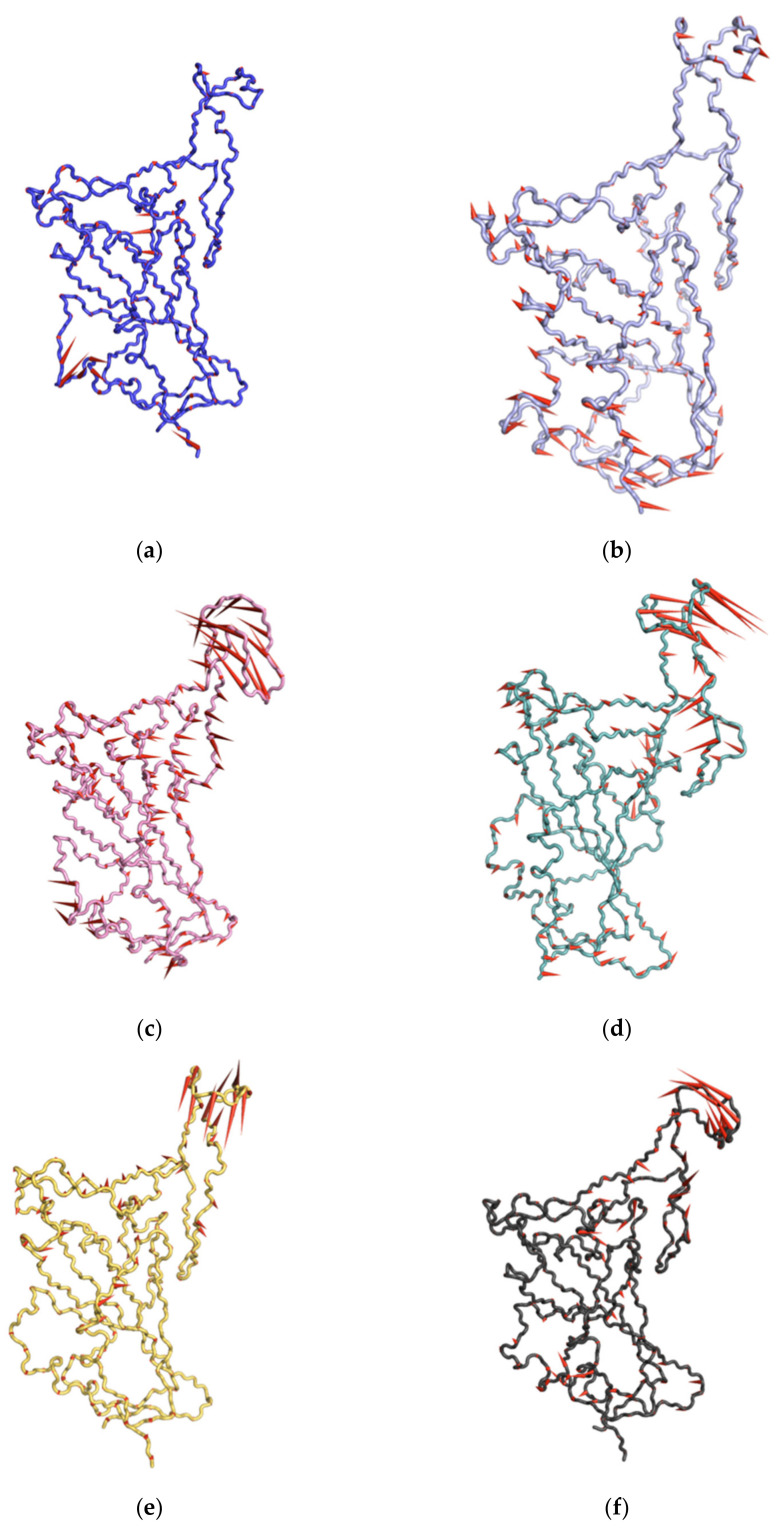
Porcupine plots showing the motion across the first principal component (PC) in WT RBD (**a**) and N501Y (**b**), L452R (**c**), S477N (**d**), N439K (**e**), and E484K (**f**) mutants. The arrows reflect the direction of the correlated motion and the extent of the motion.

Therefore, the MD trajectory of each system was inspected with PCA in order to better understand the RBD mutation effects on the major motions and conformational changes of RBD. Our analysis was restricted to RBD backbone residues in order to enhance characterization of essential space motions. Figure 5 shows 2D projection of simulation trajectories defined by the first and second eigenvectors for each mutant RBD aligned with the WT RBD of the backbone atoms. Overall, WT and all mutant RBDs showed a high overlap in conformational subspace along with eigenvector 1 (*x*-axis) and eigenvector 2 (*y*-axis) (Figure 6). Particularly, WT, N501Y, and E484K RBDs were found to sample from almost the same conformational subspace (Figure 5a,e). However, there were small differences between WT and mutant RBDs, implying a small part of the conformational subspace was not covered by WT. In N439K and L452R, there was a clear area where both could sample conformational subspace differently from WT (Figure 5b,d). However, these different conformational subspaces in the mutant RBDs were energetically unfavorable conformations based on the free energy landscape analysis below. A porcupine plot [57] of the first PC mode was used to depict the direction and extent of prominent motions in WT and mutant RBDs (Figure 6). The results showed that the prominent motions of WT RBD were located in S366–S371 and P412–D428 domains, while these motions were well distributed in S366–S371 and P384–D389 domains, as well as in loop Y495–Y508, in N501Y (which showed different conformations, as mentioned above) (Figure 6b). Finally, the prominent motion was observed at loop Y473–C489 in L452R, S477N, N439K, and E484K RBDs, which showed high flexibility over the 100-ns MD simulations (Figure 6).

### 3.8. Free Energy Landscape

**Figure 7:**
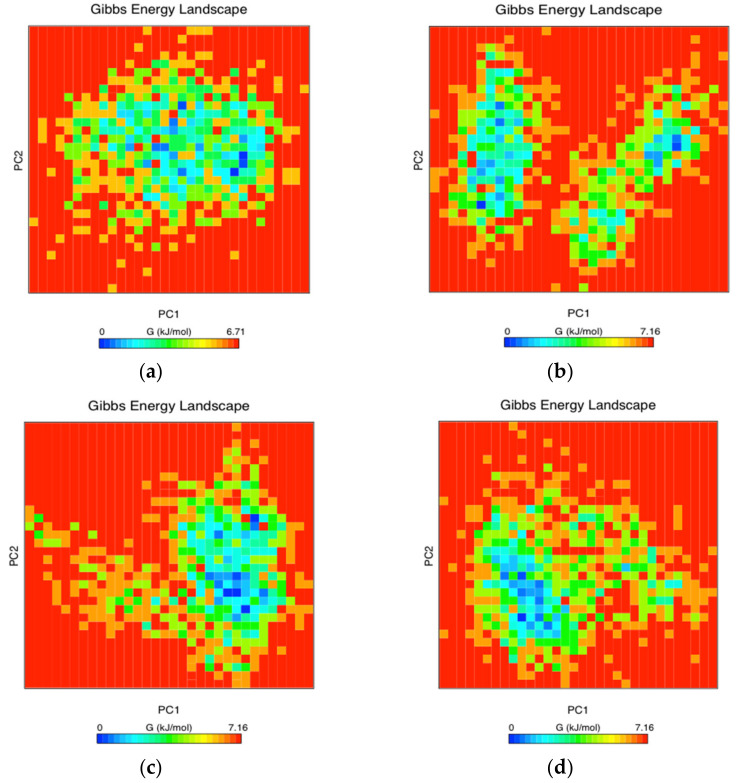

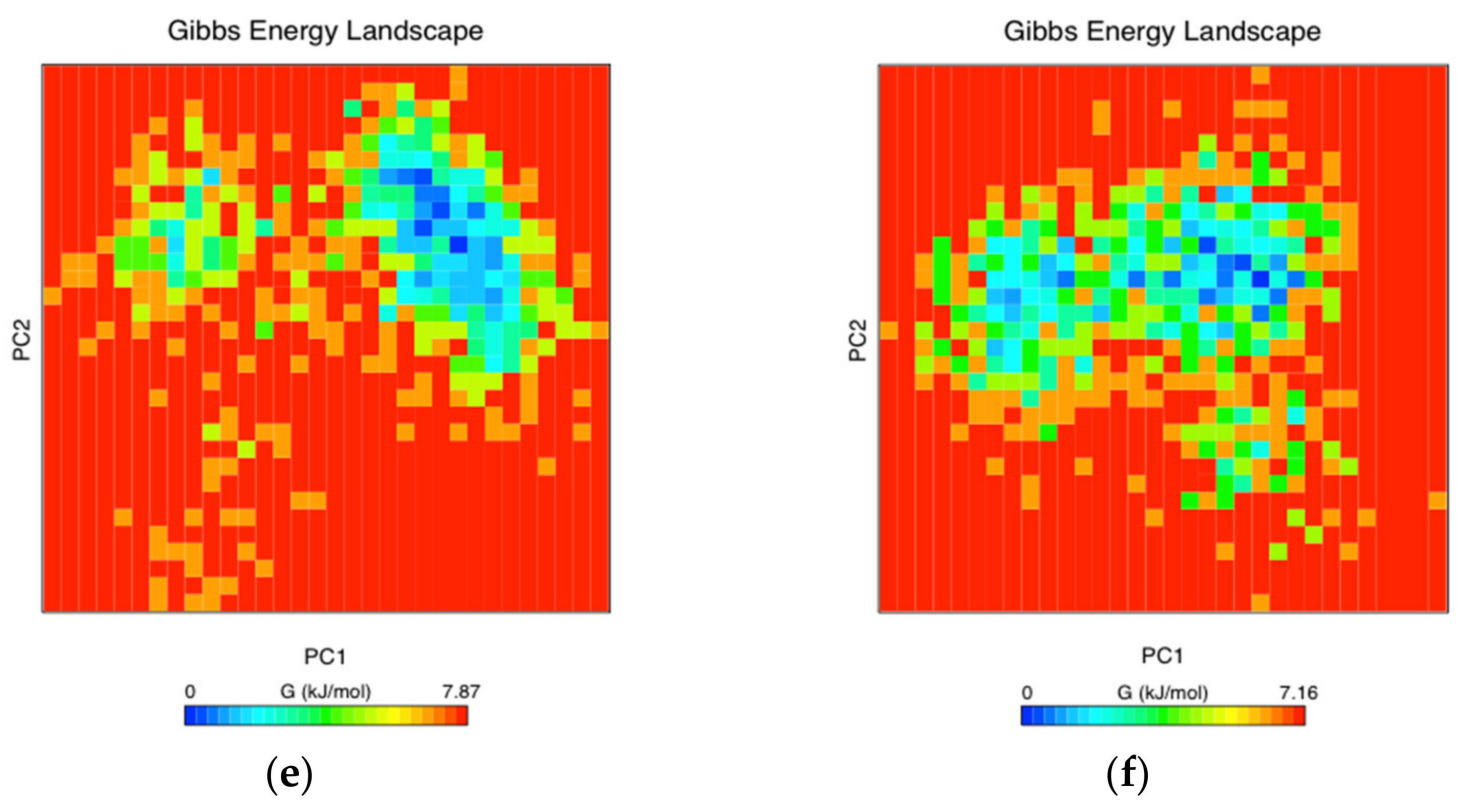
The free energy landscape (FEL) was obtained during the 100-ns MD simulations for each RBD system: WT RBD (**a**), N501Y (**b**), L452R (**c**), S477N (**d**), N439K (**e**), or E484K (**f**) RBDs.

The analysis of free energy landscape (FEL) has been used to determine lower-energy basins (minima) during MD simulations [58,59]. Here, we plotted 2D graph of the FEL using PC1 and PC2 for WT and mutants RBDs for all backbone atoms (Figure 7). The plot showed energetically favorable and unfavorable RBD conformations colored with dark blue and yellow spots, respectively. Clearly, PC1 and PC2 motion modes of mutant RBDs spanned larger ranges than WT RBD; this suggested that RBD mutations affect the WT RBD conformations (Figure 7). Additionally, all RBDs showed local minima, except for N501Y, with apparently two minima that in a confined space suggested a lower energy barrier between two conformations.

## 4. Discussion

Mutations in proteins can affect protein conformation, folding, and stability, and can eventually influence protein–protein interactions and protein thermodynamics [60]. There are several observed mutations in the RBD of SARS-CoV-2 S glycoprotein that improve its infectivity and strengthen the viral binding interaction to ACE2 receptor [27]. These mutations compromise the neutralizing ability of anti-SARS-CoV-2 antibodies; therefore, it is necessary to study the mutation effects on the RBD structure (e.g., conformation, stability, dynamics, etc.) [24,26]. Several structural and dynamic studies at the molecular level show that SARS-CoV-2 RBDs have to adapt open conformation (also known as “up” or “standing”) to effectively bind to ACE2 receptors [8,16,61,62]. So far, mutations in non-RBD residues, such as the D614G variant, can populate RBD open conformation rather than closed conformations [63]. Most of the virulent point mutations occur in the RBM loop that directly binds hACE2 and are more prone to conformational variations; thus, these mutations may have the ability to generate a more stable complex with high binding affinity [64]. For instance, N439K, L452R, T478I, and E484D mutations on RBM have significant free energy changes, and they constitute approximately 58% of all mutations on RBD. Global data analysis shows that infectivity strengthening and virion stable mutations are on the rise (especially the frequency of S477N, N439K, V483A, and V367F), clearly indicating the natural selection of mutations with stronger transmissibility [27].

Here, we studied five critical mutant RBDs according to the RBD mutation tracking website (CovMT) [33] by utilizing the all-atom MD simulation technique. The N501Y flexibility in loop Y473–C489 of RBM was comparable to WT RBD; thus, suggesting that tyrosine mutation did not alter neither the loop Y473–C489 flexibility or the whole RBD conformational compactness. Only N501Y RBD showed different loop Y495–Y508 conformations. The mutation of alanine instead of tyrosine at 501 (i.e., N501A) shows an increase in loop Y473–C489 flexibility and conformational compactness according to a related study [61]. Moreover, the same loop Y473–C489 in SARS-CoV RBD showed a higher flexibility in comparison to SARS-CoV-2 RBD [61,62]. This suggests that the higher infectivity of the N501Y variant might be attributed to an improvement in the N501Y RBD conformation and therefore a higher affinity to ACE2 receptor. Previously, substitution mutations in the SARS-CoV-2 RBD, N501, L452, N439, E484, T470, and Q498 have been shown to enhance binding affinity for hACE2, thereby increasing infectivity and transmissibility in comparison to the natural SARS-CoV-2 [65].

On the other hand, the loop Y473–C489 flexibility was increased in L452R, N439K, and E484K RBDs, and these mutations were associated with higher infectivity and binding affinity to ACE2. This was consistent with previously reported structural analyses that showed that the RBM region has the highest flexibility [9,32,49]. However, the observed rigidity in some parts of RBDs (i.e., non-RBM regions) in N439K and E484K RBDs might compensate for the entropy penalty due to flexibility in the loop Y473–C489. Therefore, the N501Y, N439K, and E484K mutations studied in this work have insignificant changes in the overall RBD flexibility. This was indicated by the SARS-CoV-2 mutations may augment the conformational sampling to avoid the entropy cost upon interaction with ACE2 receptor. In contrast, S477N and L452R RBDs showed comparable flexibility to WT RBD in non-RBM regions but higher flexibility in the RBM regions, as well as a loose conformational compactness. Our results were consistent with a pervious study that showed that S477N has a destabilizing effect on RBD structure and therefore less prone to develop disease 

PCA, FEL, and porcupine plot results suggested that the destabilizing effect could be noticed in the loop Y473–C489 of S477N RBD as compared to WT RBD. However, the conformational sampling of energetically favorable conformations of mutant RBDs showed local minima, thereby indicating stable structures for these mutant RBDs. The COVDI-19 pandemic presents a continuing threat to global health due to its ongoing critical mutations. To conquer this pandemic, it is necessary to investigate the effects of SARS-CoV-2 mutations at all possible levels, such as structural, functional, and activity levels. Moreover, RBD mutations directly affect the ability of SARS-CoV-2 to binding to ACE2 receptor and therefore affect its infectivity. Our structural investigation of critical mutant RBDs along with WT RBD showed that RBD mutations have a direct impact on the molecular structural of SARS-CoV-2. These data might be helpful for researchers investigating antiviral agents and vaccine research and development against SARS-CoV-2, especially mutant SARS-CoV-2 viruses.

## 5. Conclusions

In this study, we performed MD simulations for five RBD mutants as well as WT RBD. SARS-CoV-2 RBD mutations can directly affect RBD conformations, especially in the RBM region. Mutations in N501Y, N439K, and E484K RBDs showed insignificant changes in flexibility. The higher flexibility in S477N and L452R RBDs did not significantly affect the conformations, since the FEL analysis showed relatively local minima for each. We identified that the essential motion of E484K, N439K, S477N, and L452R RBDs were mainly in the loop Y473–C489, which is located at the binding interface with ACE2 receptor. During the COVID-19 pandemic, it is important to investigate the SARS-CoV-2 RBD molecular structural changes associated with RBD mutations. These results can help with assessing the relationship between mutations and the structural changes of RBD. Therefore, a better understanding might be established for RBD infectivity behavior due to these critical mutations, which would be helpful to conquer critical viral mutations.

## Data Availability

Data are available within the article, Supplementary Materials or from the corresponding author upon reasonable request.

## Supplementary Materials

The following are available online at https://www.mdpi.com/article/10.3390/biom11091273/s1: Figure S1: The solvent accessible surface area (SASA), Figure S2: Number of secondary structure as a function of time, Figure S3: Dictionary of Protein Secondary Structure (DSSP), Figure S4: Number of hydrogen bonds, Figure S5: Ramachandran plot, Figure S6: ProSA analysis, and Figure S7: Verify3D plot.
