# Supplementary material for: Mutations of SARS-CoV-2 RBD May Alter Its Molecular Structure to Improve Its Infection Efficiency
Source: Biomolecules. 2021 Aug 25;11(9):1273. doi: 10.3390/biom11091273 (PMC8466379; doi:10.3390/biom11091273)
Supplement: Supplementary file 1 [file biomolecules-11-01273-s001.zip › biomolecules-1279349-supplementary.pdf]

biomolecules

Article

Mutations of SARS-CoV-2 RBD may alter its molecular structure to improve its conformation

Ahmed L. Alaofi* **and Mudassar Shahid**

Department of Pharmaceutics, College of Pharmacy, King Saud University, P.O. Box 2457, Riyadh 11451, Saudi Arabia
* Correspondence: ahmedofi@ksu.edu.sa; Tel.: +966114677364; Fax: +966114676383

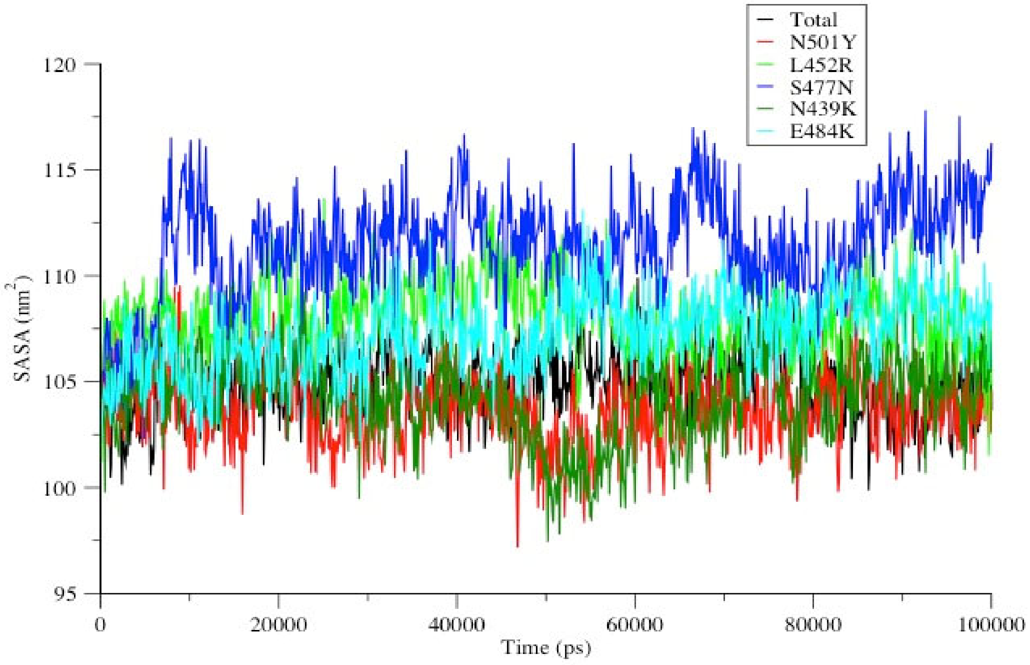

Figure S1. The solvent accessible surface area (SASA) was plotted against time (ps) for WT (black), ,N501Y (red), L452R (light green), S477N (blue), N439K (green), and E484K (cyan) RBDs.

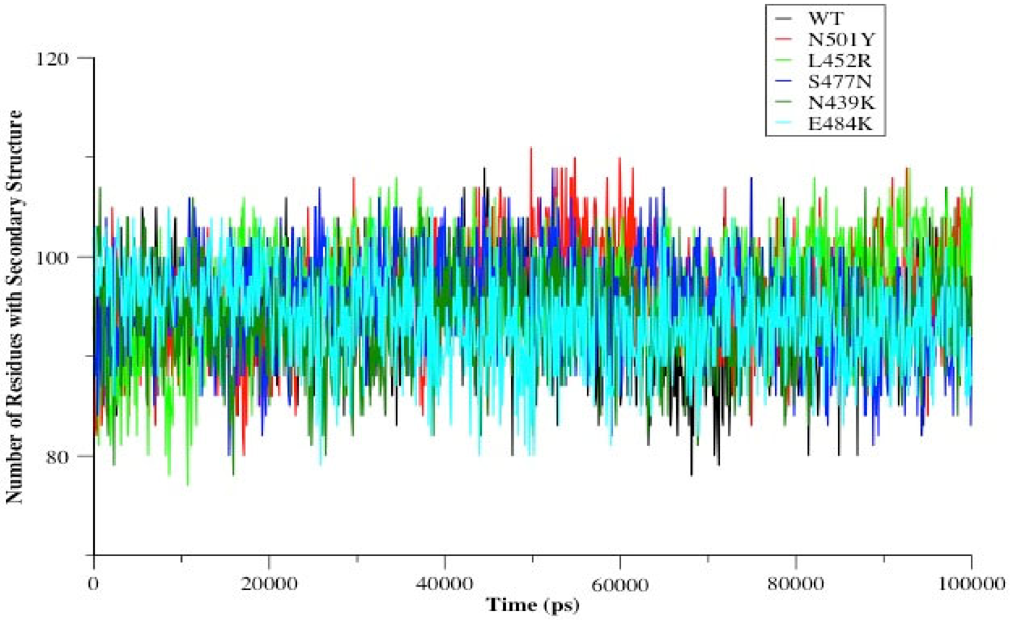

Figure S2. Number of secondary structure as a function of time (ps) for WT (black), ,N501Y (red), L452R (light green), S477N (blue), N439K (green), and E484K (cyan) RBDs during the 100-ns MD simulations.

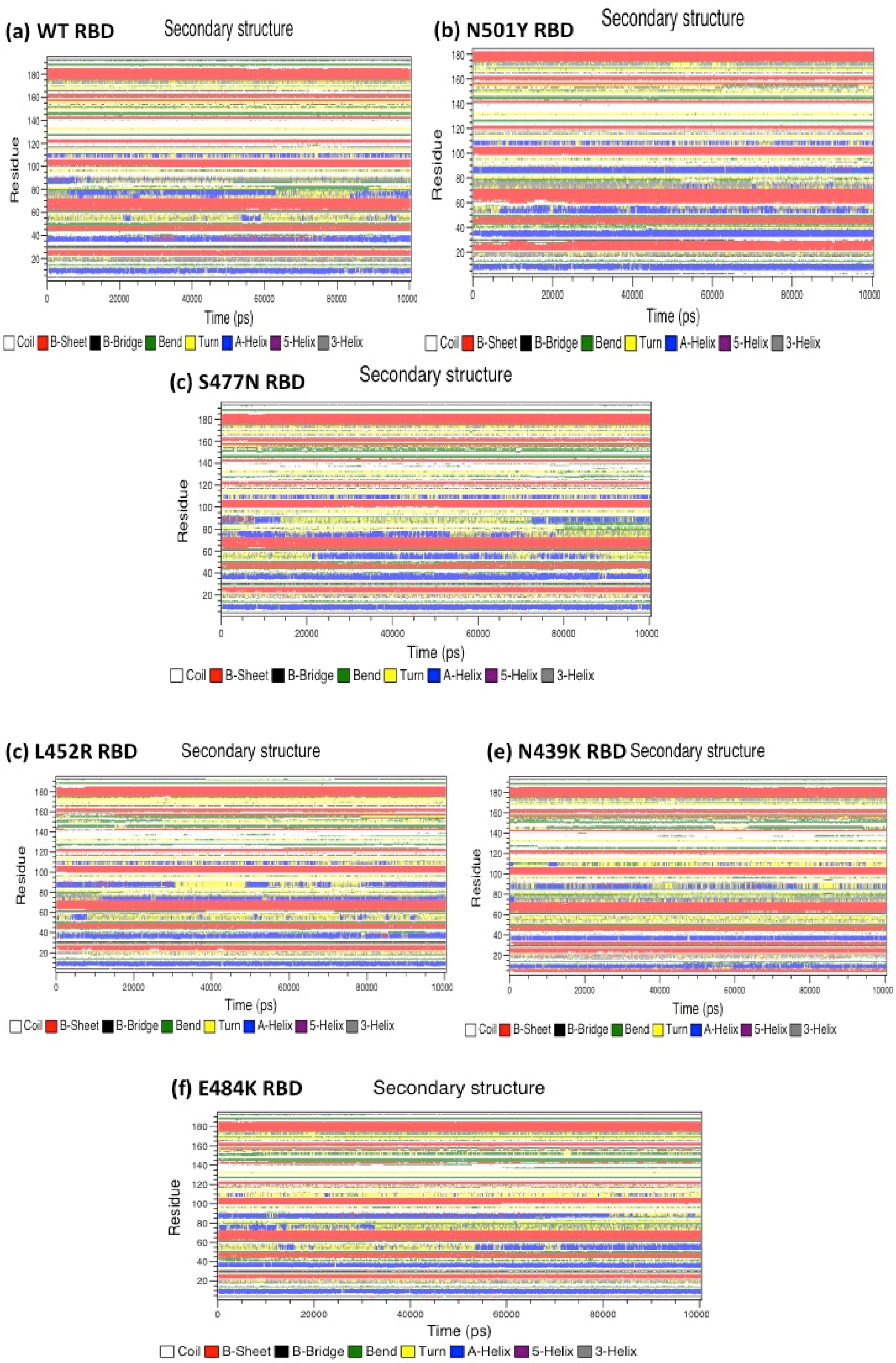

Figure S3. Dictionary of Protein Secondary Structure (DSSP) was used to monitor secondary structures changes during the 100-ns MD simulations for each system.

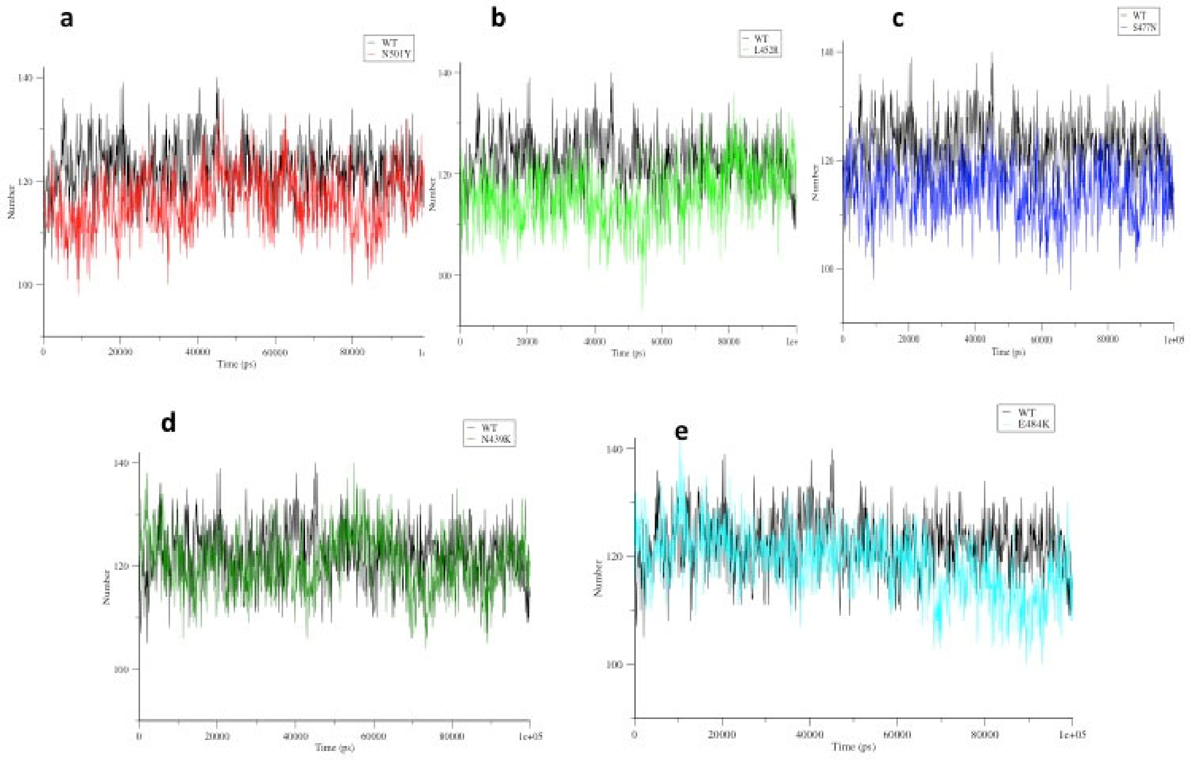

Figure S4. Number of hydrogen bonds was plotted as a function of time (ps). For comparison, number of H-bonds of WT RBD aligned with either N501Y (a), L452R (b), S477N (c), N439K (d), or E484K (e) RBDs.

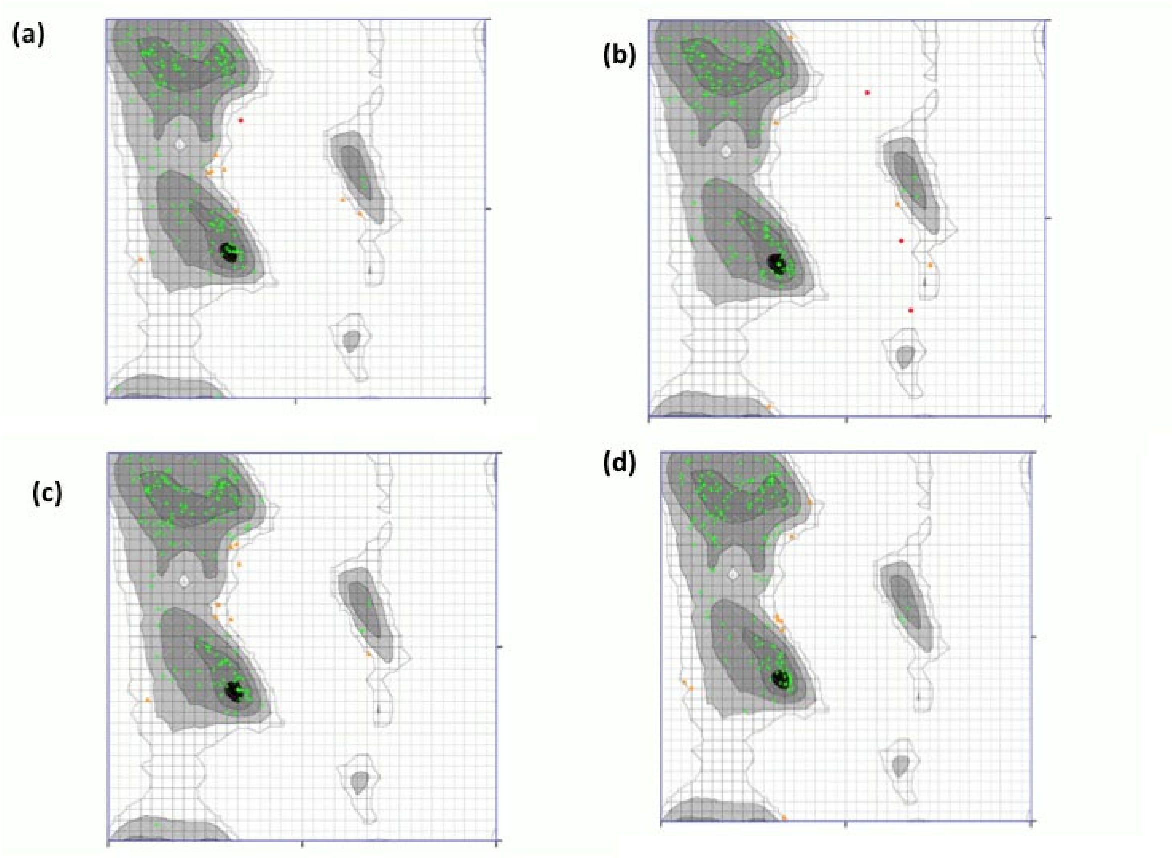

Figure S5. Ramachandran plot for modeled mutant RBDs L452R (a), S477N (b), N439K (c), and E484K (d) were obtained. The plot showed highly preferred observation more than 95% for all mutant RBDs.

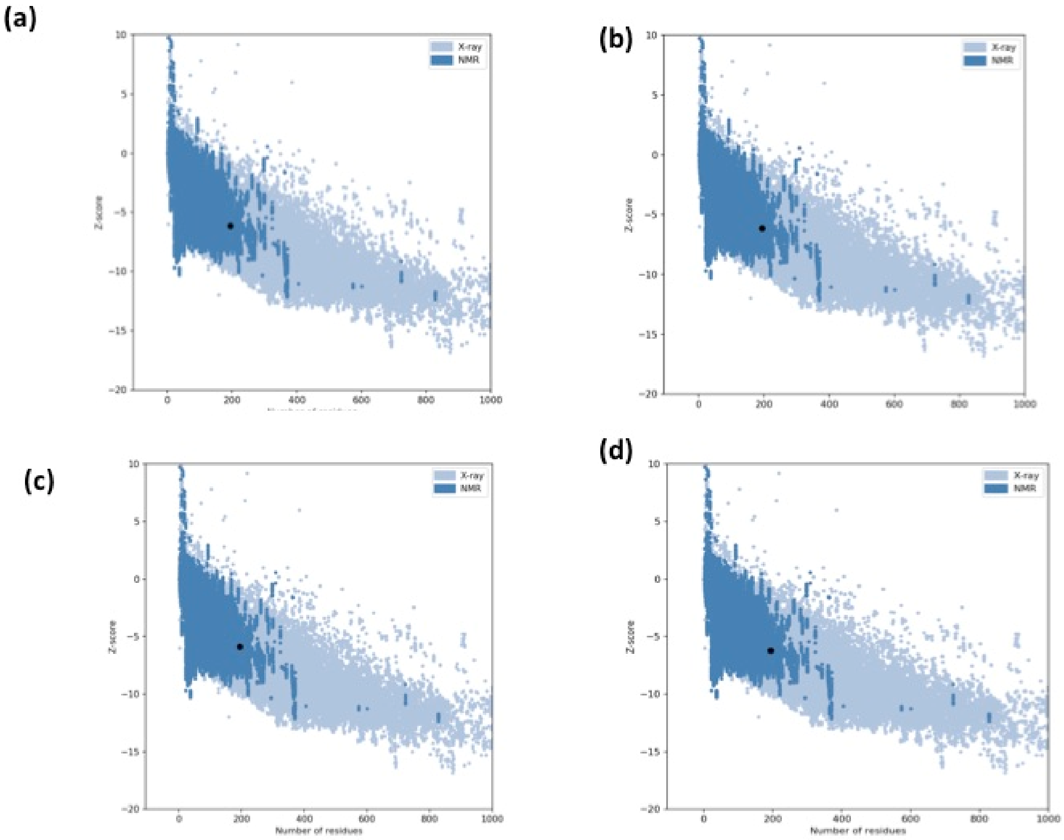

Figure S6. ProSA analysis showed z-score of -6.14, -6.13, -5.9, and -6.2 for L452R (a), S477N (b), N439K (c), and E484K (d) RBDs, respectively.

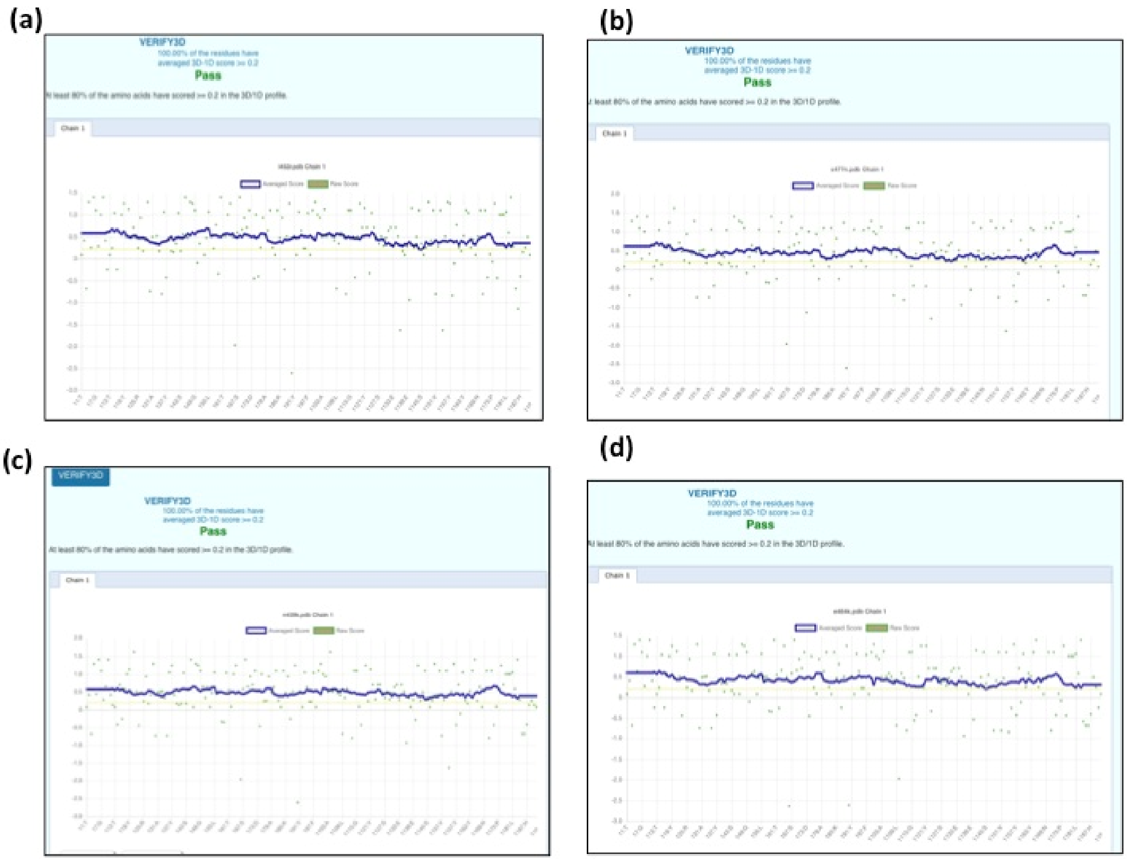

Figure S7. Verify3D was used to test the 3D structures for modeled mutant RBDs. All mutants RBDs passed the verification.
